# Dialysis membrane enclosed laccase catalysis combines a controlled conversion rate and recyclability without enzyme immobilization

**DOI:** 10.1186/s13568-020-0955-6

**Published:** 2020-01-28

**Authors:** Jie Zhang, Fukun Li, Ruiqi Wang, Xuemei Tan, Peter-Leon Hagedoorn

**Affiliations:** 1 Chongqing Engineering Research Center for Processing, Storage and Transportation of Characterized Agro-Products, College of Environment and Resources, Chongqing Technology and Business University, Chongqing, 400067 China; 2 Department of Biotechnology, Delft University of Technology, Van der Maasweg 9, 2629 HZ Delft, The Netherlands

**Keywords:** Enzyme recycling, Membrane enclosed enzymatic catalysis, Dialysis membrane, Recyclability, Laccase

## Abstract

Laccase is a versatile multicopper oxidase that holds great promise for many biotechnological applications. For such applications, it is essential to explore good biocatalytic systems for high activity and recyclability. The feasibility of membrane enclosed enzymatic catalysis (MEEC) for enzyme recycling with laccase was evaluated. The dialysis membrane enclosed laccase catalysis (DMELC) was tested for the conversion of the non-phenolic model substrate 2,2′-Azino-bis(3-ethylbenzthiazoline-6-sulfonate) (ABTS). *Trametes versicolor* laccase was found to be completely retained by the dialysis membrane during the process. The ABTS total conversion after DMELC reached the same values as the batch reaction of the enzyme in solution. The efficiency of DMELC conversion of ABTS under different process conditions including shaking speed, temperature, ABTS concentration and pH was investigated. The repetitive dialysis minimally affected the activity and the protein content of the enclosed laccase. DMELC retained 70.3 ± 0.8% of its initial conversion after 5 cycles. The usefulness of MEEC extends to other enzymes with the benefit of superior activity of an enzyme in solution and the recyclability which is normally only obtained with immobilized enzymes.
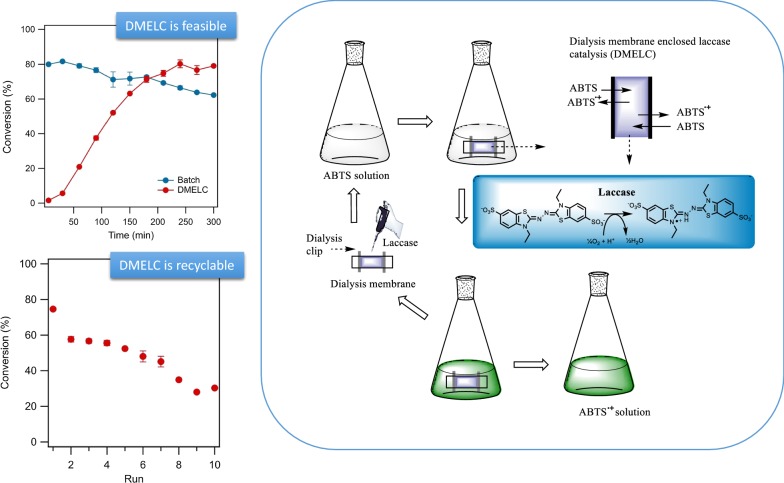

## Key points

- DMELC is a feasible biocatalysis approach.
- Kinetic and diffusion control of DMELC is possible by varying external conditions.
- Laccase recyclability with DMELC is competitive to immobilized enzyme catalysts.

## Introduction

Enzymatic bioconversion can be accomplished under mild conditions, achieving high reaction specificity and rates, and generally only requires a small amount of biocatalyst (Nguyen et al. 2014). Enzymatic processes have been developed in the food industry, fine chemicals synthesis, wastewater treatment and bioremediation (Rios et al. 2004). Batch reactions with enzymes in solution are often utilized and, most of the time, require relatively simple conditions to be controlled in the process. However, there are a certain number of disadvantages, such as the difficulty of enzyme recycling, low productivity due to product inhibition, and ineffective use of enzyme activity.

Enzyme recycling increases the catalytic productivity of the enzymes by reusing them for several batches, and thereby reduces the overall cost associated with the enzymatic bioconversion processes (Jørgensen and Pinelo 2017). Different strategies including enzyme re-adsorption on fresh solid substrate, membrane separation and enzyme immobilization have been developed to overcome, at least in part, these problems.

Enzyme re-adsorption on fresh solid substrate is a feasible approach to capture the enzyme in solution. Tu et al. (2007) indicated that the recycling of cellulases was accomplished on an ethanol pretreated Lodgepole pine (14.5% lignin) with the addition of surfactants and subsequent readsorption of desorbed cellulases onto fresh substrate. An obvious limitation of this approach is that the substrate must consist of solid particles.

Enzymes can be immobilized by adsorption or covalent binding on a solid support, reticulation or inclusion in a capsule, matrix or fiber (Rios et al. 2004). Enzyme immobilization has many advantages, such as use in continuous flow reactors, easy and selective enzyme recovery, and often long operational and storage stability (Antecka et al. 2018; Cui et al. 2018; Hu et al. 2018; Ilmi et al. 2017; Planchestainer et al. 2017; Zhang et al. 2014). Nevertheless, enzyme immobilization means a higher cost and often induces a loss of enzyme activity as high as 10–90%. Another issue is that the results of enzyme immobilization are unpredictable as there is no universal strategy towards enzyme immobilization. Therefore a lot of trial-and-error is required to find the optimal immobilization procedure for a particular enzyme and process.

Many studies have demonstrated that enzyme recycling by using membranes is an easy and feasible technique (Agustian et al. 2011; Marques et al. 2017; Nguyen et al. 2014; Possebom et al. 2014). Membranes with specific pore sizes are selected to retain or separate free or immobilized enzyme in the production medium. A typical example is the enzyme membrane reactor (EMR) providing a continuous processes in which enzymes are separated selectively by the membrane and reused. de Cazes et al. (2014) reported that the degradation of tetracycline in aqueous solutions at 20 mg/L using *Trametes versicolor* laccase during 24 h, which resulted in a conversion of 56% with EMR and only 30% with the free enzyme in batch. Marques et al. (2017) indicated that EMR can provide product removal together with reuse of enzymes and bacterial cells in the simultaneous saccharification and fermentation process for recycled paper sludge conversion into lactic acid. Although membrane fouling can be a problem for long term operation, EMR can be operated at steady state and saves a significant amount of enzyme usage during the process (Wei and Chiang 2009).

Dialysis membranes with different molecular weight cut off (MWCO) values can be applied to capture an enzyme. Bednarski et al. (1987) indicated that membrane enclosed enzymatic catalysis (MEEC), in which the enzyme in soluble form is enclosed in a (commercially available) dialysis membrane, can be utilized for the enzyme catalyzed reactions in organic synthesis. The enzyme can be retained by a specific dialysis membrane whereas the substrate can pass the membrane freely by diffusion. The substrate passing the dialysis membrane by diffusion can be converted by enzyme, and the product will equilibrate with the solution on the outside of the membrane. After completion of the reaction, the enzyme in the dialysis membrane can be recycled by centrifuging or dialysis, and also used directly without any treatment.

**Fig. 1:**
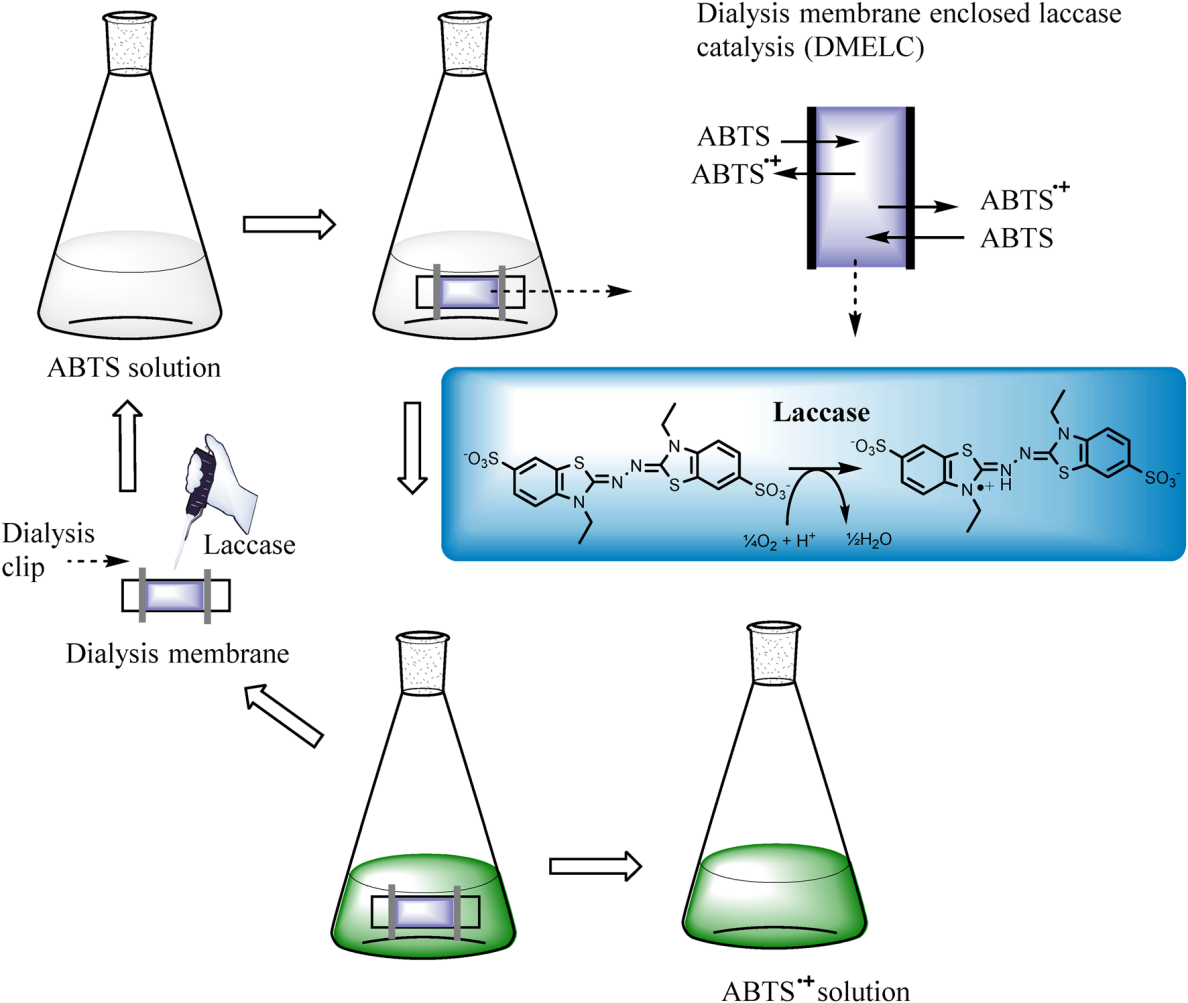
The schematic diagram of enzyme recycling using DMELC for ABTS conversion

Here we present an investigation of the feasibility of enzyme recycling by MEEC in an enzymatic bioconversion process with *T. versicolor* laccase (EC 1.10.3.2), as shown in Fig. 1. Laccase is a multi-copper-containing enzyme, that catalyzes one-electron oxidation of a range of inorganic and aromatic substances, like ortho-and para-diphenols, aminophenols and aryl diamines, with the concomitant four-electron reduction of molecular oxygen to water (Braunschmid et al. 2018; Frasconi et al. 2010; Quintanar et al. 2007; Solomon et al. 1996; Wellington et al. 2018). 2,2′-Azino-bis(3-ethylbenzthiazoline-6-sulfonate) (ABTS) was selected as a typical non-phenolic chromogenic laccase substrate. The conversion of ABTS by dialysis membrane enclosed laccase catalysis (DMELC) was evaluated. The effects of shaking speed, temperature, pH and substrate concentration on DMELC were determined, and the recyclability of DMELC is reported.

## Materials and methods

### Materials

Dialysis membrane with MWCO 3500 Da, diameter 16 mm, flat width 25 mm, was supplied by Medicell Membranes Ltd (London, UK) and stored in a dry, well ventilated room at room temperature. It was washed in warm distilled water for the removal of glycerin prior to use. Laccase from *T. versicolor* with 23.1 U/mg of enzyme activity using catechol as substrate at pH 5 and 25 °C was purchased from Fluka (Buchs, Switzerland), CAS No. 80498-15-3. Bicinchoninic acid (BCA) Protein Assay Kit was obtained from Interchim Uptima (Montluçon, France). Sodium chloride and acetic acid were purchased from Thermo Fisher Scientific (Landsmeer, The Netherlands). Sodium acetate anhydrous was purchased from Merck chemicals (Amsterdam, The Netherlands). All other chemicals were of reagent grade and were purchased from Sigma Aldrich (Zwijndrecht, The Netherlands).

### The general procedure of DMELC

The DMELC procedure used for ABTS conversion is schematically depicted in Fig. 1. The wet dialysis membrane (tubing), 7 cm of length, was closed at one end (1.5 cm) with a dialysis clip. Laccase (2.0 mg) was added in the membrane filled with 3 mL of buffer solution. Then the other end of membrane (1.5 cm) was also closed. ABTS was dissolved in the same buffer solution as inside the membrane. The ABTS solution (95 mL) was introduced in a conical flask. The membrane containing laccase was added to the flask and finally, the flask was incubated in an Innova 44 controlled shaker (Eppendorf, Nijmegen, The Netherlands) for the catalytic reaction. A fresh dialysis membrane was used to maintain the same conditions in all experiments except for the recyclability experiments.

### Determination of laccase diffusion behavior across the dialysis membrane

The laccase diffusion across the dialysis membrane was measured. The dialysis membrane was loaded with 2.0 mg laccase dissolved in 3 mL of 0.01 M potassium phosphate buffer, 10 mM NaCl (pH 5.8). The filled membrane was introduced into a conical flask containing 95 mL of 0.01 M potassium phosphate buffer, 10 mM NaCl (pH 5.8). The flask was incubated on a shaker for approximately 48 h at 30 °C, 180 rpm. Samples were taken from the solutions on the outside and the inside of membrane and analyzed for laccase activity and protein concentration.

### Influence of shaking speed, temperature, substrate concentration and pH on DMELC

ABTS conversion by DMELC was measured at different shaking speeds from 60 rpm to 180 rpm. The detailed steps were similar to those described above. Laccase (2.0 mg) was added in the dialysis membrane contained 3 mL 0.01 M potassium phosphate buffer, 10 mM NaCl (pH 5.8). ABTS (95 mL 100 μM) dissolved in 0.01 M potassium phosphate buffer, 10 mM NaCl (pH 5.8) was used. The flasks were incubated at 30 °C in a shaker. ABTS conversion by a batch reaction with the same enzyme amount in solution was performed under the same conditions. Furthermore, the effect of temperature on DMELC was tested in the range of 25 °C to 45 °C and 180 rpm.

ABTS concentrations of 50, 100, 200, 400 and 800 μM were selected to investigate the effect of substrate concentration at a pH of 5.8 (0.01 M potassium phosphate buffer, 10 mM NaCl), 30 °C and 180 rpm based on the results.

The influence of pH was investigated in the pH range of 3.6 to 7.0 at ABTS concentration of 100 μM, 30 °C and 180 rpm. Acetate buffer (0.01 M) with 10 mM NaCl was used at pH at 3.6 and 4.5, and potassium phosphate buffer (0.01 M) with 10 mM NaCl was selected for pH 5.8 and 7.0.

All experiments were performed in duplicate. Samples (100 μL each time) were taken from the dialysate solution every 30 min to determine the ABTS^•+^ concentration spectrophotometrically over a period of 300 min.

### Recyclability of laccase in DMELC

Based on the results from the tests described above, the optimal conditions (temperature, 30 °C, shaking speed, 180 rpm, ABTS concentration, 100 μM, pH, 4.5) for ABTS conversion by laccase were used to assess the recyclability of MEEC. The reaction using the same dialysis membrane enclosed laccase was repeated 10 consecutive times as described above. After each reaction, the dialysis membrane enclosed laccase was removed from the conical flask into a centrifuge tube, and it was centrifuged with a centrifuge (5810R, Eppendorf) at 4 °C, 2500 rpm for 120 min. Then the dialysis membrane enclosed laccase was filled with the same buffer again and placed into a conical flask contained fresh ABTS solution. A sample was taken to measure the ABTS^•+^ concentration at the end of each catalytic reaction (180 min). One control reaction without ABTS and another control without ABTS and dialysis membrane but with the same procedure were tested for protein concentration and enzyme activity.

### ABTS^•+^ concentration determination

For the analysis of the oxidation product from ABTS, the UV–Vis spectrum (200 nm to 800 nm) of ABTS, its cation radical, ABTS^•+^ and laccase (21 μg/mL) was measured with a UV–Vis spectrophotometer (Cary 60, Agilent). Based on the tested absorbance spectra, the detection wavelength was set at 420 nm for ABTS^•+^, and the molar extinction coefficient ε_420_ = 36 mM^−1^ cm^−1^ was used (Johannes and Majcherczyk 2000). Each sample (100 μL) was diluted with 900 μL buffer prior to the determination of the ABTS^•+^ concentration.

### Protein determination

The protein concentration was determined using a commercial protein quantitation kit (BC Assay kit, Interchim Uptima). The prepared samples were mixed with the BCA reagent and incubated at 37 °C for 30 min, and the absorbance at 562 nm was measured with a UV–Vis spectrophotometer (Cary 60, Agilent). Samples (100 μL each sample) were taken and kept frozen at -21 °C prior to measurement.

### Laccase activity assay

The laccase activity assay was performed according to Mishra and Kumar (2007) with some modifications using a Cary 60 UV–Vis spectrophotometer (Agilent). The assay solution (1.05 mL) contained 950 μL 1.0 mM ABTS (0.01 M potassium phosphate buffer pH 5.8 containing 10 mM NaCl), 10–100 μL sample and 0–90 μL 0.01 M potassium phosphate buffer pH 5.8 containing 10 mM NaCl. The initial rate during the first 60 s at 30 °C was measured and used to calculate the activity. One unit (U) of laccase activity was defined as the amount of laccase that oxidizes 1 μmol ABTS per min. The specific activity was expressed in U per milligram laccase (U/mg).

## Results

### The feasibility of DMELC

**Fig. 2:**
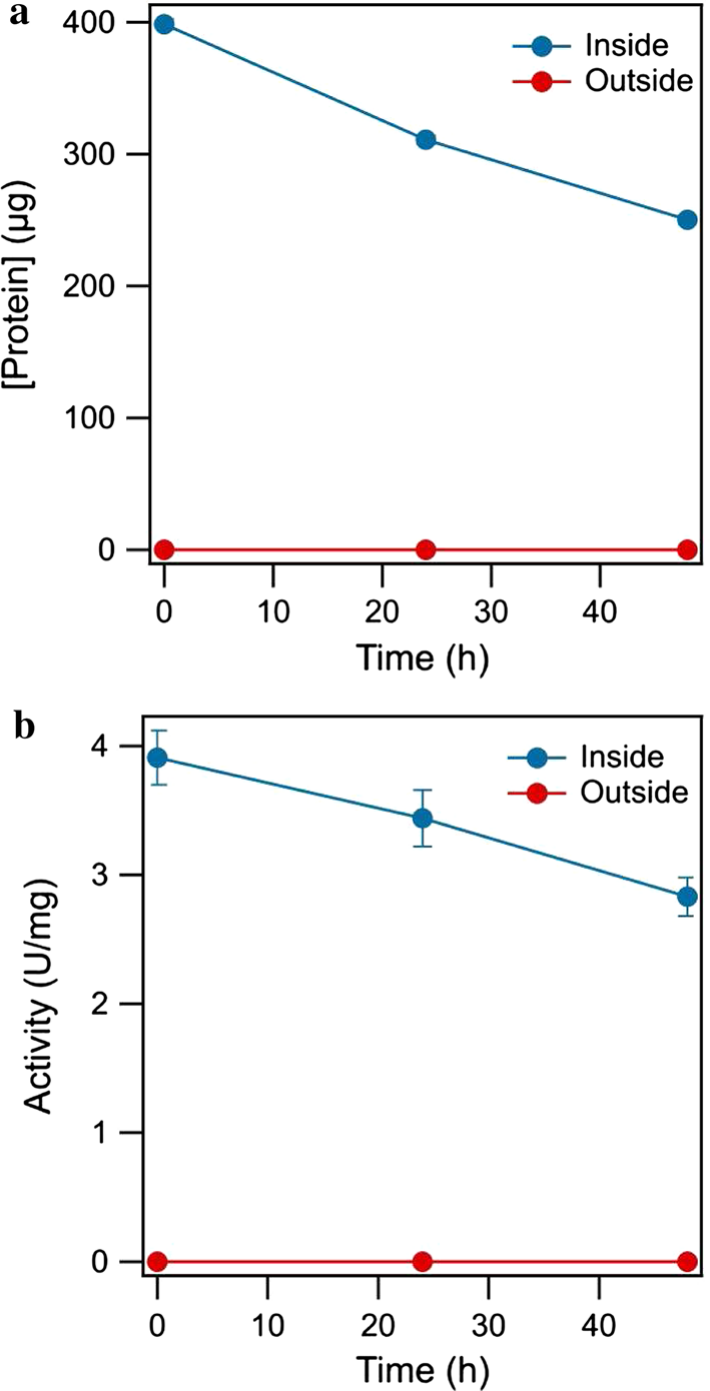
Diffusion behavior of laccase over the dialysis membrane, **a** protein and **b** laccase activity (temperature, 30 °C, shaking speed, 180 rpm, pH, 5.8, 2.0 mg laccase weight)

Dialysis is a molecular separation procedure based on selective diffusion to change the concentration or composition of solutes. The most common application of dialysis is for the removal of unwanted small molecules such as salts, reducing agents, or dyes from larger macromolecules such as proteins, DNA, or polysaccharides (Berg et al. 2007). Laccase activity and total protein were measured to evaluate the diffusion behavior of laccase over the dialysis membrane, as shown in Fig. 2. The MWCO of the dialysis membrane, 3500 Da, was selected on the basis of the molecular weight of ABTS (512.7 Da) and *T. versicolor* laccase (50–60 kDa). No laccase activity and protein concentration was detected in the solution outside the dialysis membrane over a period of 48 h. The total protein remained 62.8 ± 0.8% of the initial protein inside of the dialysis membrane, and laccase specific activity remained 72.4 ± 3.8% compared to the initial activity. A laccase solution kept in a water bath at 25 °C lost about 50% of its activity within the first 5 days, and the loss of activity was almost complete after 16 days due to microbial degradation (Mai et al. 2000). The loss of activity coincided with a reduction of the total protein level.

**Fig. 3:**
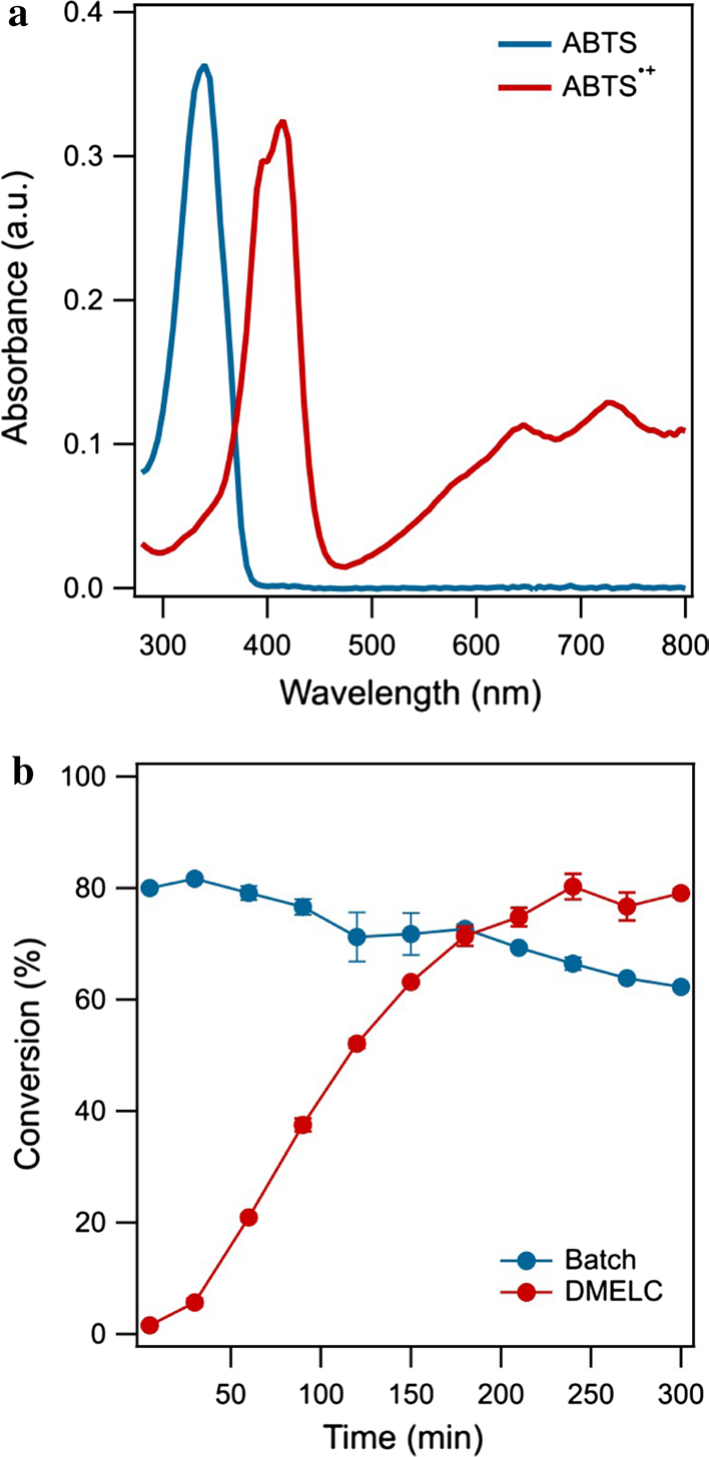
**a** Absorbance spectra of ABTS and its cation radical, ABTS^•+^. **b** Time-course of ABTS^•+^ production by DMELC and batch reaction of laccase in solution (temperature, 30 °C, shaking speed, 180 rpm, ABTS concentration, 100 μM, pH, 5.8, 2.0 mg laccase)

To confirm that the absorbance changes at 420 nm could be utilized to monitor the product ABTS^•+^, the UV–Vis absorbance spectrum was compared with that of the substrate ABTS as shown in Fig. 3a. The maximum absorption wavelength of ABTS and ABTS^•+^ was 340 nm and 420 nm, respectively. Moreover, laccase in the concentration used did not give a significant absorbance in the range from 300 to 800 nm (not shown), corresponding with the study of Zhang et al. (2012). The absorbance of laccase in the visible range was negligible in the enzyme concentration range used.

The dialysis membrane tube containing laccase was placed in the ABTS solution to measure the conversion of ABTS by DMELC. As expected ABTS can pass the dialysis membrane and react with free laccase due to the low molecular weight of ABTS. The blue-green oxidation product ABTS^•+^ was produced inside of the dialysis membrane, and diffused to the outside of the membrane. The batch reaction of laccase in solution (not enclosed) as shown in Fig. 3b, resulted in nearly complete conversion of ABTS into ABTS^•+^ in the first 5 min and the ABTS^•+^ concentration decreased slowly after 30 min. Using DMELC ABTS converted to ABTS^•+^ steadily and achieved the highest product concentration at 240 min which was equal to the product concentration at 5 min for the free enzyme batch reaction.

### Effects of shaking speed and temperature on DMELC

It is well known that enzymatic efficiency is affected by the substrate and product concentration and many physical parameters, such as pH, ionic strength, and temperature. At the same time the utilization of the dialysis membrane will infer mass transfer limitation which will affect the rate of the enzyme reaction by controlling the substrate and product concentrations to which the enzyme is exposed as well as the rate of product formation on the outside of the membrane. Therefore, the effect of these factors on DMELC was studied.

**Fig. 4:**
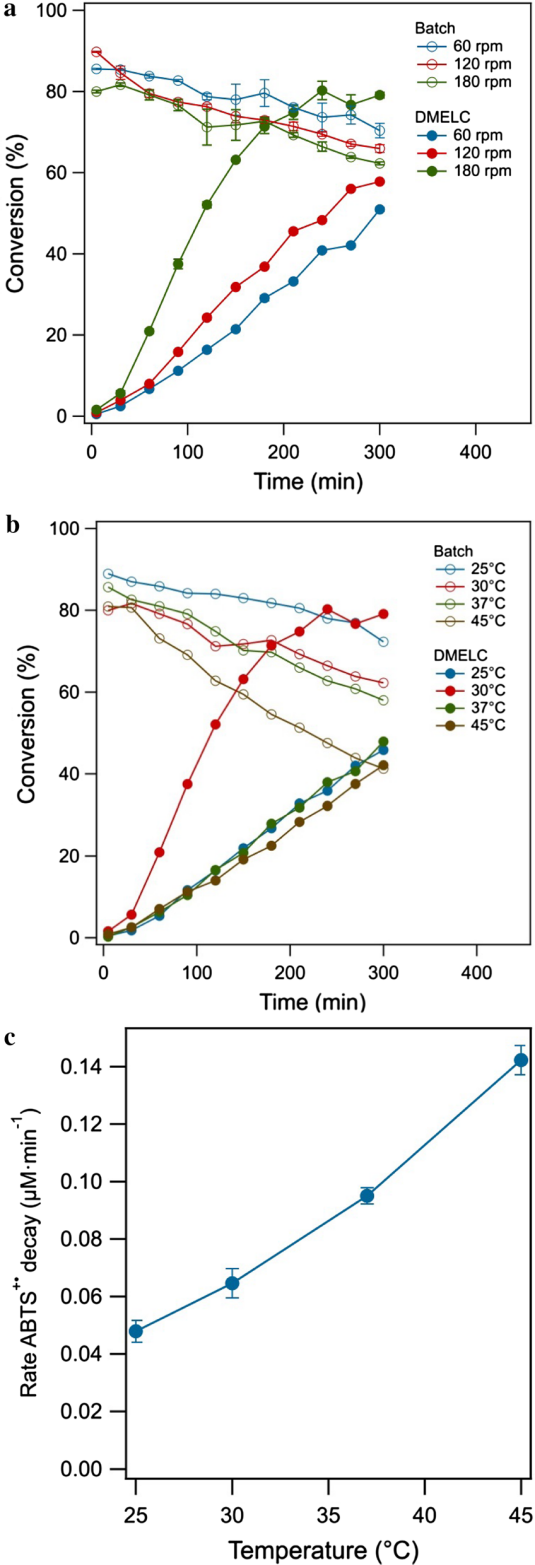
**a** Time-course of ABTS^•+^ production by DMELC and batch reaction of laccase in solution at different shaking speeds (temperature, 30 °C, ABTS concentration, 100 μM, pH, 5.8, 2.0 mg laccase). **b** Time-course of ABTS^•+^ production by DMELC and batch reaction of laccase in solution at different temperatures (shaking speed, 180 rpm, ABTS concentration, 100 μM, pH, 5.8, 2.0 mg laccase). **c** Temperature dependence of the rate of ABTS^•+^ degradation

The exchange of substrate (ABTS) and product (ABTS^•+^) over the dialysis membrane affecting enzymatic reaction in the process can be influenced by shaking or stirring the solution. In Fig. 4a, ABTS^•+^ (oxidation product) concentration over time using DMELC and a batch reaction of laccase in solution at different shaking speeds were compared. For the batch reaction ABTS^•+^ reached the peak concentration after 5 min incubation at 120 rpm and the conversion for the first 5 min was lower at 60 and 180 rpm. At all three shaking speeds there is a similar linear decline of the product concentration over time from 5 to 300 min. For DMELC, ABTS^•+^ concentration was almost the same value at 5 min and increased over time with three shaking speeds. As stated above the rate of product formation was dependent on the shaking speed. The rate of product formation strongly increased from 120 to 180 rpm (Fig. 4a),

The influence of temperature on the production of ABTS^•+^ for both DMELC and batch reactions of laccase in solution is shown in Fig. 4b. Four temperatures were studied 25, 30, 37 and 45 °C, higher temperatures were excluded because the optimum temperature range of this laccase has been reported to be within 15–50 °C (Chea et al. 2012; Delanoy et al. 2005). In the batch reaction, the highest product concentration was obtained at 25 °C within 5 min incubation. At higher temperatures a significant linear decline of ABTS^•+^ was observed. The rate of ABTS^•+^ degradation increased with temperature (Fig. 4c), which is consistent with Cano et al. (1998) who reported that ABTS^•+^ became highly unstable above 35 °C. In DMELC, at different temperatures the same ABTS^•+^ concentration was produced in the initial 5 min. The ABTS^•+^ concentration increased linear between 25 °C and 37 °C at least from 30 to 180 min of DMELC. The optimal rate of product formation was observed at 30 °C, and similar rates were observed for 25 and 37 °C and the lowest rate was observed at 45 °C.

### Effects of substrate concentration and pH on DMELC

**Fig. 5:**
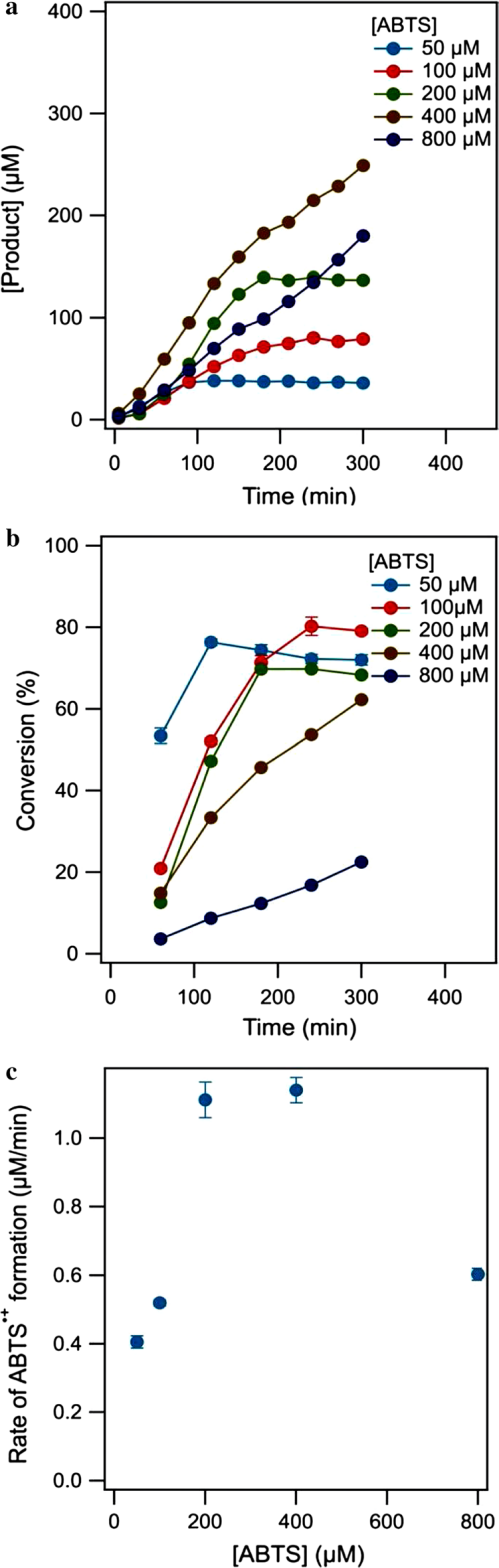
The effect of ABTS concentration on ABTS^•+^ production (**a**), the conversion of ABTS (**b**), and the rate of product formation (**c**) by DMELC (temperature, 30 °C, shaking speed, 180 rpm, pH, 5.8, 2.0 mg laccase)

DMELC using different substrate concentrations showed that the plateau values of product reached with 50, 100 and 200 μM substrate represent full conversion of the substrate (Fig. 5a). The relationship between conversion and concentration of ABTS given in Fig. 5b shows that at higher ABTS concentration more time was needed to achieve full conversion. The highest conversion, 80.3 ± 2.3%, was obtained using an ABTS concentration of 100 μM and after 240 min. The rate of product formation was dependent of the substrate concentration, as shown in Fig. 5c. The product formation rate increased with the ABTS concentration up to 400 μM.

**Fig. 6:**
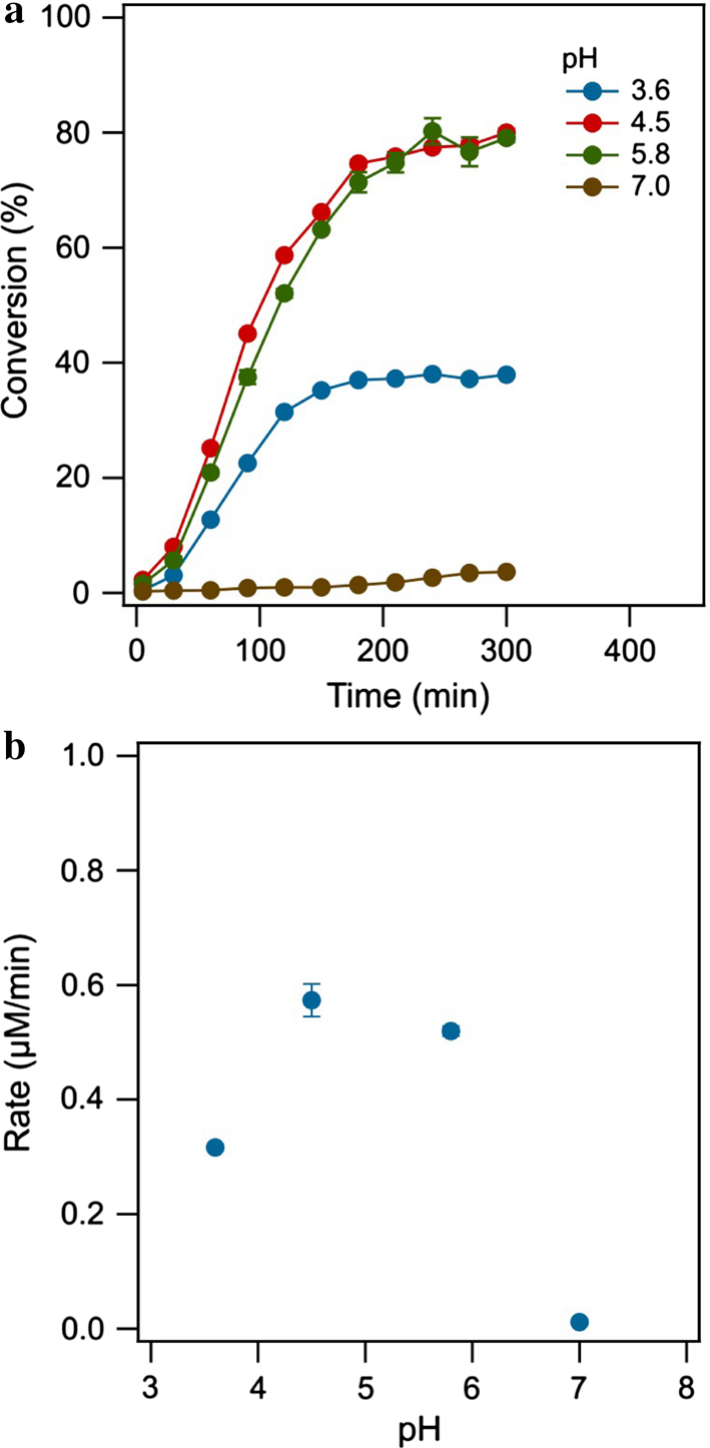
The effect of pH on the conversion of ABTS (**a**) and the rate of product formation (**b**) by DMELC (temperature, 30 °C, shaking speed, 180 rpm, ABTS concentration, 100 μM, 2.0 mg laccase)

The ABTS conversion by DMELC was investigated in the pH range of 3.6 to 7.0 (Fig. 6). *T. versicolor* laccase has been reported to exhibit optimal enzyme activity at acidic pH of 3–5 (Chea et al. 2012; Plagemann et al. 2011). The rate of product formation was found to be pH dependent. The rate of product formation at pH 4.5 was higher than at the other three pH values during the whole reaction process. At pH 4.5 the rate was the highest, although at pH 5.8 the same final conversion was reached.

### Recyclability of DMELC

**Fig. 7:**
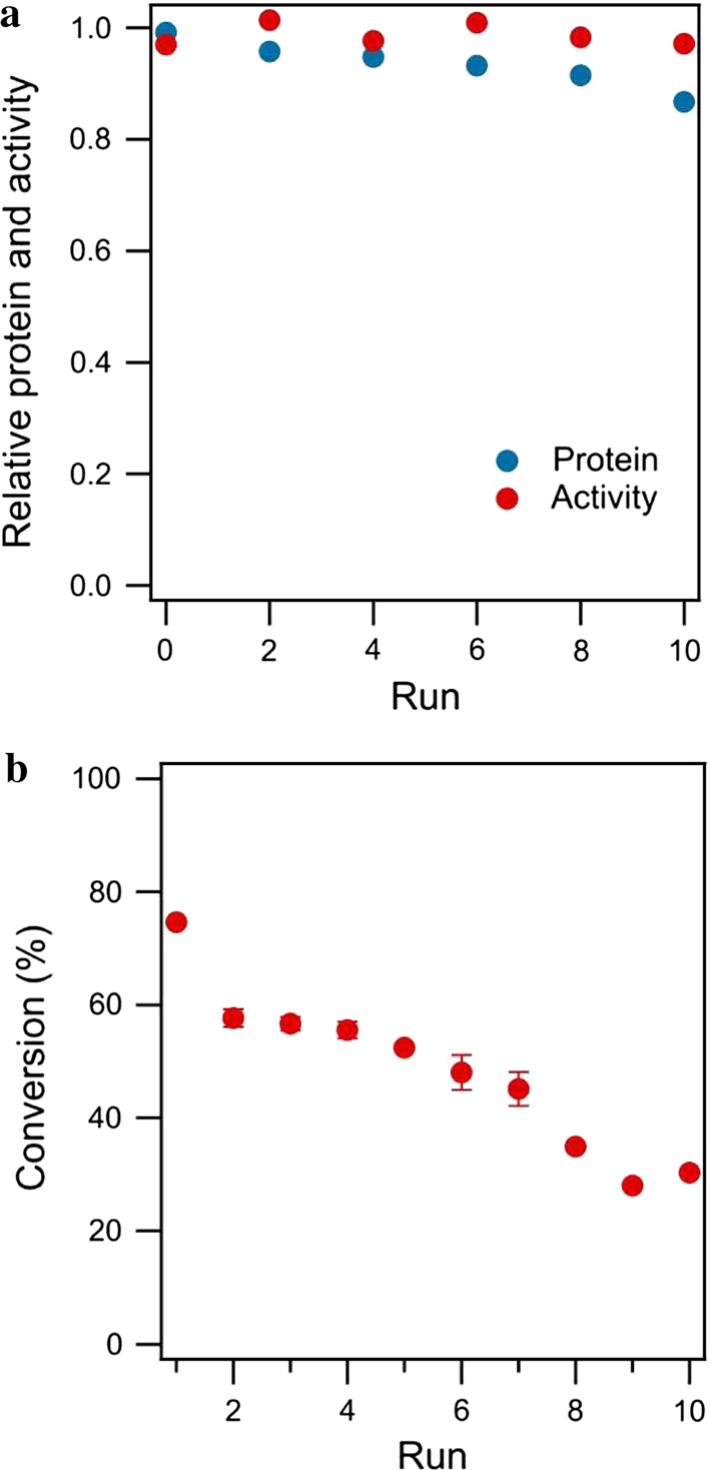
**a** Effect of the repetitive dialysis on the protein concentration and activity of laccase. **b** Recyclability of DMELC for ABTS conversion (temperature, 30 °C, shaking speed, 180 rpm, ABTS concentration, 100 μM, pH, 4.5, 2.0 mg laccase, time of each DMELC, 180 min)

As described above MEEC is a feasible strategy for a bioconversion process with laccase and ABTS. In order to evaluate diffusion behavior and laccase stability after repetitive dialysis cycles, the dialysis membrane containing laccase was incubated 10 repeated runs without ABTS. The determination of laccase activity and protein concentration proved to be difficult when the enzyme was already exposed to ABTS. To verify that the results of laccase activity and protein concentration took place only due to dialysis, control experiments containing only laccase (without the dialysis membrane) were performed in parallel. Laccase was successfully retained because protein and laccase activity could not be detected outside of the dialysis membrane during the whole run. Figure 7a shows the protein concentration and activity of the laccase after dialysis cycles normalized for the parallel control experiments. A slight decrease of total protein and a stable activity level were found. The protein concentration and laccase activity in the control experiments decreased due to the microbial degradation. The half-life of laccase was well over 20 dialysis runs.

For any industrial application, the recyclability of an (immobilized) enzyme is a key factor for its cost-effective use (Cui et al. 2018; Santos et al. 2015). Therefore, the recyclability of DMELC in several operating cycles was explored (Fig. 7b). After an initial drop in the total conversion after the first cycle of DMELC, the total conversion decreased gradually after each cycle. The total conversion after cycle 5 was 70.3 ± 0.8% of the conversion after cycle 1.

## Discussion

### DMELC is a feasible biocatalysis approach

The results demonstrated that laccase was completely retained in the dialysis membrane due to the high molecular weight of laccase and that no significant leakage occurred within the timeframe of the experiments. Sterile conditions need to be employed to prevent the loss of protein and activity of the laccase during multiple days of operation.

The ABTS^•+^ radical, produced in the batch reaction, was found to decrease in concentration over time. ABTS^•+^ is relatively stable, but it can react enzymatically and non-enzymatically with other compounds, which may account for the observed decrease (Romay et al. 1996). Franco et al. (2014) indicated that the ABTS^•+^ concentration of a solution stored in the dark at room temperature decreased during the first 18 h, and subsequently it was stable over the following 42 h.

This showed that DMELC can achieve the same high total conversion as the batch reaction of laccase in solution, however after a much longer time due to mass transfer limitations caused by the dialysis membrane. Clearly the dialysis membrane controlled the rate of ABTS production by DMELC. Similarly to the batch reaction with free enzyme, the ABTS^•+^ concentration also decreased after reaching a plateau value. Therefore, the dialysis membrane with a suitable MWCO can successfully retain laccase and allow diffusion of the substrate ABTS in and the product ABTS^•+^ out, resulting in a controlled DMELC reaction. Earlier studies have shown that a membrane enclosed laccase could be used to decolorize synthetic dyes (Katuri et al. 2009; Wong and Yu 1999).

### Kinetic and diffusion control of DMELC by varying shaking speed, temperature, pH and substrate concentration

The dependency of the DMELC conversion rate on the shaking speed confirms that the rate of product formation was limited by the mass transfer rate over the dialysis membrane. The mass transfer rate over the dialysis membrane can be further accelerated by increasing the shaking rate or by stirring both the inner or outer solution (Ogston 1960).

The apparent lag in product formation during the first 5–15 min that can be seen in the DMELC reactions likely represents the time required for initial equilibration of the solutions on the outside and inside the dialysis membrane. It takes time before the laccase inside the membrane is exposed to the same concentration of substrate as outside of the membrane, and to equilibrate the formed product concentration on both sides.

Temperature has three major effects on DMELC: (1) the effect on enzymatic kinetics; (2) the effect on enzyme stability (Delanoy et al. 2005), and (3) the effect on the ABTS substrate and product diffusion rate over the dialysis membrane. Increasing temperature leads to a higher diffusion coefficient and facilitates ABTS and ABTS^•+^ permeability (Liu et al. 2014). Nevertheless, increasing the temperature above 30 °C resulted in the significant decrease of product concentration due to instability of the product as shown in the batch reaction (Tonin et al. 2016).

The DMELC efficiency was strongly dependent on the substrate concentration in the range from 0 to 800 μM. The K_M_ for ABTS is in the range of 0.1 mM, so the rate during DMELC was dependent on the ABTS concentration according to Michaelis–Menten kinetics. Above a certain rate the mass transfer rate became limiting, which is what happened at 200 μM substrate. Under these conditions the mass transfer rate over the dialysis membrane limited the product formation rate. However, the reaction rate at 800 μM substrate was significantly lower than at 400 μM. We attribute the lower rate at high substrate concentration to product inhibition and O_2_ limitation of the membrane enclosed enzyme. The one electron oxidation of ABTS by laccase requires 1/4 oxygen molecule, which means most of the dissolved oxygen would be depleted after full conversion of 800 μM ABTS. The rate of oxygenation of the solution would then limit the rate during DMELC.

DMELC was found to be diffusion controlled between pH 4.5 and 5.8. At pH 3.6 the total conversion reached a plateau at 40% conversion which likely reflects the instability of the enzyme at this pH. At pH 7 the conversion of ABTS was only 4% which is consistent with the known pH dependence of activity for this enzyme. Under these conditions the rate of product formation is not limited by diffusion over the membrane, but by the performance of the enzyme (Delanoy et al. 2005).

A shaking speed of 180 rpm, a temperature of 30 °C, a substrate concentration of 100 μM and pH 4.5 were found to be optimal for DMELC under the conditions that have been tested. Further control over the rate and total conversion of the process can be achieved by further improving the mass transfer rate over the dialysis membrane.

### Laccase recyclability during DMELC is competitive with immobilized enzyme catalysts

The results have shown that laccase is stable during the repetitive dialysis process. The DMELC recyclability is comparable to the recyclability of immobilized laccase reported by Li et al. (2018), which indicated that the conversion of the substrate hydroquinone remained at approximately 70% by graphene aerogel-Zr-MOF with immobilized laccase after five catalyst reuse cycles. However, the relative conversion dropped below 50% after 8 cycles and 39.5 ± 0.7% at the tenth run. A study by Xia et al. (2018) showed that laccase immobilized on polyethylenimine modified amine-functionalized Fe_3_O_4_ nanoparticles retained 85% of its initial activity after six consecutive operation cycles. Laccase-immobilized dendritic nanofibrous membranes maintained a high bisphenol A conversion rate of up to 79% even after four filtration cycles (Koloti et al. 2018). Xu et al. (2017) reported that laccase immobilized on nano-copper incorporated electrospun fibrous membrane still had a 2,4,6-trichlorophenol conversion rate of 65.9% after being used seven times. Although the recyclability of DMELC does not outperform certain immobilized laccase catalysts, MEEC was an effective and straightforward strategy to obtain a highly active and recyclable biocatalyst, which also facilitates downstream processing.

Thus, DMELC demonstrates the potential for enzymatic bioconversion and simultaneously enzyme recycling and easy downstream processing. However, additional research is necessary to further characterize and model the precise contribution of enzyme kinetics, substrate and product diffusion over the membrane, enzyme stability involved in MEEC, which would result in a highly predictable laccase biocatalyst.

## Data Availability

The authors confirm that the data supporting the conclusions are given in the article. Raw data was stored on secure servers at the Delft University of Technology and is available upon request to the corresponding author PLH.

## Abbreviations

ABTS: 2,2′-Azino-bis(3-ethylbenzthiazoline-6-sulfonate)
Da: Dalton
DMELC: dialysis membrane enclosed laccase catalysis
EMR: enzyme membrane reactor
MEEC: membrane enclosed enzymatic catalysis
MWCO: molecular weight cut-off
